# Cannabis and Depression: A Twin Model Approach to Co-morbidity

**DOI:** 10.1007/s10519-017-9848-0

**Published:** 2017-05-02

**Authors:** M. Smolkina, K. I. Morley, F. Rijsdijk, A. Agrawal, J. E. Bergin, E. C. Nelson, D. Statham, N. G. Martin, M. T. Lynskey

**Affiliations:** 1 National Addiction Centre, Institute of Psychiatry, Psychology, and Neuroscience, King’s College London, Addiction Sciences Building, 4 Windsor Walk, SE5 8BB, London, UK; 2 Centre for Epidemiology and Biostatistics, Melbourne School of Population and Global Health, The University of Melbourne, Melbourne, VIC Australia; 3 Social, Genetic & Developmental Psychiatry Centre, Institute of Psychiatry, Psychology, and Neuroscience, King’s College London, London, UK; 4 Department of Psychiatry, Washington University School of Medicine, St. Louis, MO USA; 5 Virginia Institute for Psychiatric and Behavioral Genetics, Virginia Commonwealth University, Richmond, VA USA; 6 Faculty of Arts, Business and Law, School of Social Sciences, University of the Sunshine Coast, Sippy Downs, QLD Australia; 7 Genetic Epidemiology, QIMR Berghofer Medical Research Institute, Brisbane, QLD Australia

**Keywords:** Co-morbidity, Major depressive disorder, Cannabis use disorder, Twin model, Genetics

## Abstract

Cannabis use disorder (CUD) co-occurs with major depressive disorder (MDD) more frequently than would be expected by chance. However, studies to date have not produced a clear understanding of the mechanisms underlying this co-morbidity. Genetically informative studies can add valuable insight to this problem, as they allow the evaluation of competing models of co-morbidity. This study uses data from the Australian Twin Registry to compare 13 co-morbidity twin models initially proposed by Neale and Kendler (Am J Hum Genet 57:935–953, 1995). The analysis sample comprised 2410 male and female monozygotic and dizygotic twins (average age 32) who were assessed on CUD and MDD using the SSAGA-OZ interview. Data were analyzed in OpenMx. Of the 13 different co-morbidity models, two fit equally well: *CUD causes MDD* and *Random Multiformity of CUD*. Both fit substantially better than the *Correlated Liabilities* model. Although the current study cannot differentiate between them statistically, these models, in combination, suggest that CUD risk factors may causally influence the risk to develop MDD, but only when risk for CUD is high.

## Introduction

Major depressive disorder (MDD) and Cannabis use disorder (CUD) are co-morbid (see Degenhardt et al. 2012) and highly relevant from a public health perspective. According to the latest Global Burden of Disease study in 2013, MDD was the 2nd leading cause of disability in the world (Ferrari et al. 2013), and the World Health Organization is now citing it as the leading one (WHO 2016). Heavy cannabis use is linked to several adverse health outcomes (Hall and Degenhardt 2009; Hall 2015), and is the most commonly used illicit drug (Agrawal and Lynskey 2014). The relationship between MDD and CUD is poorly understood, although individuals with co-morbid mental health and substance use disorders are particularly difficult to treat (Kessler 2004). Developing a greater understanding of the relationship between CUD and MDD is therefore important in order to help reduce the prevalence of both conditions through the efficient prevention and treatment of co-morbid cases.

Cross-sectional studies of general and clinical populations consistently show that CUD and MDD co-occur at a rate greater than chance (see Degenhardt et al. 2012 for a review). For instance, an epidemiological study of 43,093 US citizens showed that individuals with mood disorders (MDD, dysthymia, mania, hypomania) had 3.9 (95% CI 2.8–5.3) times higher odds of meeting criteria for lifetime cannabis abuse and dependence (Martins and Gorelick 2011). An epidemiological survey of 25,113 Canadian citizens reported that rates of past-year cannabis dependence among individuals who met 12 month MDD criteria were over 7.25 times higher compared to those who did not (Patten et al. 2015). Rates of past-year abuse were almost 3.6 times higher (Patten et al. 2015). Similar results have been found in clinical samples. For example, a recent study based on the Norwegian patient registry including 2,659,966 individuals, reported that levels of ICD-10 depressive illness were almost 3.9 times higher among individuals with CUD (12.85%), compared to the general population (3.3%, Nesvåg et al. 2015).

There have been several attempts, including longitudinal and genetic studies, to explain this pattern of co-morbidity. Most longitudinal studies have looked at the relationship between MDD and cannabis use, rather than CUD. Studies investigating non-heavy cannabis use have not been able to establish a clear causal link in either direction (e.g. Lev-Ran et al. 2014; Feingold et al. 2014; Cougle et al. 2015; Danielsson et al. 2016). However, heavy cannabis use is more consistently and more strongly associated with MDD. In a meta-analysis Lev-Ran et al. (2014) found a moderate (OR 1.62, 95% CI 1.21–2.16) increase in risk of developing depressive disorders following heavy cannabis use. The strongest statistically significant relationships have been observed between clinical levels of cannabis use and MDD, with cannabis abuse preceding MDD (OR 4.00, 95% CI 1.23–12.99, Bovasso 2001) and CUD preceding MDD (OR 2.54, 95% CI 1.40–4.60, Marmorstein et al. 2012). However, there is also some evidence of a bidirectional relationship, from baseline CUD to incident MDD (OR 1.78, 95% CI 1.17–2.71), as well as baseline MDD to incident CUD (OR 2.28, 95% CI 1.28–4.05; Pacek et al. 2013), while other studies have found no significant relationships (Harder et al. 2008; Feingold et al. 2014).

Overall, causal influences, mainly from CUD to MDD, may be present, but clear conclusions are precluded by the heterogeneity among studies, particularly in terms of the control for confounding factors. Feingold et al. (2014) found that cannabis users and non-users differed significantly on age, gender, household income and marital status. An early study by Fergusson and Horwood (1997) also demonstrated differences on a large number of factors, including childhood adversities, social disadvantage, contact with peers who engaged in substance use or delinquent behaviors, and psychological adjustment problems. Establishing causality in either direction therefore still requires further evidence.

Previous twin studies investigating this co-morbidity have been scarce, and offered mixed evidence. In a discordant twin study by Lynskey et al. (2004), there was a significant genetic correlation between cannabis dependence and MDD (men, *r* = 0.44, 95% CI = 0.17–1.00; women, *r* = 0.69, 95% CI 0.30–1.00), but cannabis dependence was not a unique causal factor for MDD. Among MZ twins discordant for cannabis dependence, the dependent twins did not have greater odds for MDD (OR 1.16, 95% CI 0.64–2.17). Lynskey et al. (2004) also found no evidence of causality in the opposite direction (OR 1.38, 95% CI 0.55–3.42). In contrast, Lin et al. (1996) reported that MZ twins with MDD were more likely to be cannabis dependent (OR 2.3, 95% CI 1.1–4.7), compared to their co-twins without MDD. Differences in results between these studies might be explained by differences in their samples. Lin et al.’s (1996) analysis sample, i.e. twins discordant for lifetime MDD, comprised 234 male veterans twin pairs, while Lynskey et al. (2004) examined a sample of 156 twin pairs from the general population, including both male and female same-sex twins. Additionally, Fu et al. (2002) found that antisocial personality disorder explained 62% of the genetic correlation between CUD and MDD in a multivariate twin study.

This mixed evidence suggests that the exact nature of the relationship between CUD and MDD warrants further study. One possibility to comprehensively investigate competing models of co-morbidity is to fit Neale and Kendler’s (1995) 13 co-morbidity models, which were based on the work of Klein and Riso (1993). Each model and each class of models makes different assumptions about the etiological mechanisms that lead to the co-morbidity. Four broad classes are examined: single liability, independent liability, multiformity and correlated liabilities. No other twin model approach examines such a large variety of model classes. If the co-morbid form arises from a *single liability* shared by CUD and MDD, the diagnostic boundary between MDD and CUD may have been artificially drawn, and they could be alternate forms of the same disorder. Alternatively, liability to the co-morbid form may be entirely *independent*: co-morbidity arises due to a third disorder, unrelated to the pure forms of MDD and CUD. *Multiformity* models suggest that the risk factors for CUD and MDD are unrelated, but once certain thresholds on the liability of one disorder are crossed, the risk of symptoms of the other disorder increases sharply. In other words, MDD and CUD influence each other in a discontinuous way, only once certain levels of risk are reached. In contrast, *correlated liabilities* models assume that liabilities between two disorders are related continuously, and etiological factors overlap. Any change in risk for one disorder is accompanied by a change in risk for the other disorder, whether this is due to shared risk factors or causality. In addition, the models test whether the co-morbidity observed in the population has occurred by chance.

The NK comorbidity models have been used to examine the relationship between a range of other substance-use phenotypes (Agrawal et al. 2004, 2007, 2010). Twin models of co-morbidity would also be a useful tool to study the relationship between cannabis involvement and depression (Agrawal and Lynskey 2014), as both MDD (e.g. Sullivan et al. 2000; Kendler et al. 2006a) and cannabis dependence (Lynskey et al. 2002; Verweij et al. 2010) are influenced by genetic factors.

To date, no study has examined all 13 models with respect to these phenotypes, although previous longitudinal and twin studies have produced conflicting findings regarding the relationship between MDD and CUD. Therefore, the current study aimed to fit all 13 NK co-morbidity models to examine the relationship between CUD and MDD in a cross-sectional sample of 2410 Australian twins born between 1972 and 1979.

## Methods

### Participants

From a sample of 4131 twin pairs included in the Australian Twin Registry, 3824 twins and non-twin siblings born between 1972 and 1979 were interviewed on cannabis use, related drug use and other psychopathology (see Lynskey et al. 2012 for further details of the sample). The analyses presented in this paper were conducted on twins only and required complete data from each twin pair for both phenotypes. Consequently, 2410 individual twins were included in the analysis sample: 565 (396 female, 169 male) complete MZ pairs and 640 (298 female–female, 118 male–male and 224 female–male) complete DZ twin pairs. The mean age of the sample was 32 years.

### Measures

#### SSAGA-OZ interview

Computer-assisted telephone interviews based on the Australian version of Semi-Structured Assessment of the Genetics of Alcoholism (SSAGA-OZ; Bucholz et al. 1994) were used to assess twins on several variables. The SSAGA-OZ has been widely used in family studies of alcohol dependence and collects detailed information on patterns of DSM-IV (American Psychiatric Association 2000) symptomatology across a range of mental health and substance use disorders. Assessments of these disorders, including MDD and CUD, have been shown to have good reliability and validity (Bucholz et al. 1994). In order to keep the measures comparable to current literature, items were coded as close to DSM-5 (American Psychiatric Association 2013) as the available information allowed. Although coding the phenotypes as binary variables reduces statistical power, the co-morbidity models were developed for and can currently only be fitted to binary data.

*Cannabis Use Disorder:* Participants were assessed on DSM-IV Cannabis Abuse/Dependence criteria. Abuse items included “hazardous use”, “social/interpersonal problems related to use”, “neglecting major roles to use” and “legal problems”. Dependence items included “tolerance”, “using larger amounts or using longer”, “repeated attempts to quit or control use”, “much time spent using”, “physical or physiological problems related to use”, and “activities given up to use”. In addition, an assessment of cannabis withdrawal was available (see Verweij et al. 2013). However, no assessment of “craving”, a criterion introduced in DSM-5, was available for this sample. CUD was coded as a binary phenotype. To approximate DSM-5 criteria, individuals were coded as 1 (“affected”) if they reported at least 2 symptoms of DSM-5 CUD, except for “craving”. The remaining participants were coded as 0 (“unaffected”), whether or not they reported a lifetime history of cannabis use. The “legal problems” criterion was removed from DSM-5 and therefore was not included in our definition of CUD.

*Major Depressive Disorder:* Participants were assessed on the following DSM-IV symptoms of MDD: “depressed or irritable mood”, “loss of pleasure”, “change in appetite or weight”, “change in sleep”, “energy loss or fatigue”, “change in psychomotor activity”, “feelings of guilt or worthlessness”, “difficulty concentrating or making decisions”, and “suicidal ideation”.

MDD was also coded as a binary phenotype. The participant was coded as 1 (“affected”) if they experienced 5 or more of the above symptoms for over 2 weeks, including depressed/irritable mood or loss of pleasure. Participants were not coded as having MDD if they met the following exclusion criteria: (i) their symptoms did not affect functioning in any area of life, or (ii) they occurred within 2 months of bereavement, (iii) within 1 month of using tranquilizers, blood pressure medication or steroids, or (iv) just after having used illegal drugs, alcohol or tobacco. As there have been no major changes in the diagnostic criteria for MDD between DSM-IV and DSM-5, the coding was representative of DSM-5 MDD.

### Statistical models

A summary of all models can be found in Table 1. Each model makes different assumptions about the way in which co-morbid cases arise. A detailed discussion of each model can be found in Neale and Kendler (1995) and Rhee et al. (2004).

**Table 1:** Summary and interpretation of Neale and Kendler (1995) models of co-morbidity

Nr.	Model	Sub-models	Description applied to CUD and MDD
1	Alternate forms		Single liability: after crossing a common threshold, some develop CUD, some MDD. MDD and CUD are alternate forms of the same disorder
2	Three independent disorders		Pure forms are *unrelated* disorders. Three independent liabilities for CUD, MDD and co-morbid CUD with MDD
	Multiformity		The liabilities for CUD and MDD are *unrelated*. CUD discontinuously increases the risk of MDD symptoms, and vice versa when thresholds are crossed. Random multiformity assumes one, extreme multiformity two thresholds
3	Random multiformity (RM)		Assumes a single threshold within one disorder (e.g. CUD), above which the risk to develop symptoms of the other disorder (e.g. MDD) suddenly increases. This model allows for *both* disorders to increase the risk of symptoms of the respective other
4		RM of MDD	Being above the threshold for MDD risk leads to a sudden increase in risk for symptoms of CUD, even when below the threshold for CUD
5		RM of CUD	Being above the threshold for CUD risk leads to a sudden increase in risk for symptoms of MDD, even when below the threshold for MDD
6	Extreme multiformity (EM)		There are two distinct thresholds for both disorders. Crossing the 1st threshold leads to the pure form of a disorder. The 2nd threshold allows for individuals with high amounts of risk factors. Individuals will be at increased risk for symptoms if they are above the 2nd threshold (at increased risk) for either disorder
7		EM of MDD	Being above the 1st threshold for MDD risk only leads to MDD. A proportion of high-risk individuals with MDD (above the 2nd threshold) develop CUD symptoms, even when below the 1st threshold for CUD
8		EM of CUD	Being above the 1st threshold for CUD risk only leads to CUD. A proportion of high-risk individuals with CUD (above the 2nd threshold) have MDD symptoms, even when below the 1st threshold for MDD risk
9	Correlated liabilities		Correlation between latent genetic and environmental influences on CUD and MDD gives rise to co-morbidity
10		Reciprocal causation	Liability for CUD has causal influence on liability to experience MDD, and vice versa
11		Unidirectional: MDD to CUD	Liability to experience MDD has causal influence on liability for CUD
12		Unidirectional: CUD to MDD	Liability to experience CUD has causal influence on liability for MDD
13		Chance	Co-morbid CUD and MDD occur due to chance alone

Because both phenotypes were coded as binary variables, the foundation of each co-morbidity model was a normal liability threshold model, which is based on the multifactorial theory of inheritance (Falconer 1965).

Similar to widely used liability threshold models, all models estimate genetic (A), shared (C) and non-shared environmental (E) factors. D was not estimated in the current sample, because the difference between MZ and DZ correlations indicated an ACE model. However, there are several important differences between models:

1. The models differ in the number of liability distributions they assume. For instance, the *Alternate Forms* model assumes that both phenotypes arise from *one* distribution of liability. In contrast, the *Three Independent Disorders* model assumes that there are *three* underlying liability distributions. Two of those give rise to the pure forms of the phenotypes, and one gives rise to the co-morbid form.
2. The models differ in the way in which the above-mentioned liabilities produce the phenotype. For example, in the *Alternate Forms* model, an individual develops co-morbid CUD and MDD by crossing the threshold on the shared liability distribution. However, in the *Three Independent Disorders* model, an individual can develop CUD and MDD if they cross the threshold on the CUD-specific and MDD-specific distribution at the same time, or if they do so on the liability distribution for the co-morbid form.
3. One model, the *Extreme Multiformity* Model, also differs from all others in the number of thresholds it assumes. Under the assumptions of this model, each liability distribution has two thresholds. If an individual crosses the first threshold, they only develop the pure form of a disorder. Crossing the second threshold means that the individual develops the co-morbid form. Consequently, co-morbidity arises if an individual crosses the first threshold on both liability distributions, the second threshold on one liability distribution (e.g. CUD), and/or the other distribution (e.g. MDD).

### Data analysis

Data analysis was conducted using OpenMx (Neale et al. 2016) for R statistical software (R Core Team 2014). The input to each model was a frequency table, which summarized the number of twin pairs fitting into 10 MDD-CUD co-morbidity categories (see Table 2). Each twin pair member was assigned to one of four disease state categories: MDD but no CUD (i.e. 1 0), no MDD, but CUD (i.e. 0 1), both MDD and CUD (i.e. 1 1), and neither (i.e. 0 0). Thereafter, twin disease states were combined (i.e. 0 0 0 1). Although there are 16 different combinations of co-twin disease states, information about twin order was disregarded to avoid low cell counts. For instance, “0 0 1 0” (see Table 2) is a category that contains cases where twin 1 only (i.e. 1 0 0 0) or twin 2 only (i.e. 0 0 1 0) was affected by MDD. Subsuming all replicating disease states resulted in ten categories.

**Table 2:** Number of twin pairs in co-morbidity status categories

	Twin 1		Twin 2		MZ	DZ
	MDD	CUD	MDD	CUD		
1	0^a^	0	0	0	298	277
2	0	0	0	1^b^	28	73
3	0	0	1	0	114	145
4	0	0	1	1	17	35
5	0	1	0	1	16	10
6	0	1	1	0	6	21
7	0	1	1	1	16	23
8	1	0	1	0	47	33
9	1	0	1	1	12	18
10	1	1	1	1	11	5
Total					565	640

Used as input for all co-morbidity models

^a^0 unaffected

^b^1 affected

For every model, the number of twin pairs expected in each of the 10 categories was based on the assumptions of the model. Maximum likelihood estimation was used to minimize the difference between the observed number of cases in each co-morbidity category and the expected number according to the model. A chi-squared goodness-of-fit (χ^2^) test compared these observed and expected values, and indicated model fit. The p value of the χ^2^ test was used to reject models whose predicted data was significantly different from the observed data. The best fitting and most parsimonious model was chosen based on the Akaike Information Criterion (AIC; Akaike 1987). According to Burnham and Anderson (2002) an AIC difference of 3 and over indicates that the model with the lower AIC has substantially more support.

## Results

In the analysis sample, 15.4% (11.9% of females, 22.6% of males) met criteria for lifetime CUD and 26.1% (29.5% of females, 19.2% of males) met criteria for lifetime MDD. Females had a significantly higher prevalence of MDD (OR 1.77, 95% CI 1.44–2.17) and lower prevalence of CUD (OR 0.46, 95% CI 0.37–0.58). CUD was almost twice as frequent in individuals with lifetime MDD (24.3%), compared to those without (12.3%). The odds ratio, adjusted for sex and age, was 2.66 (95% CI 2.10–3.37).

A conditional logistic regression of MZ twin pairs discordant for CUD showed that MZ twins with CUD had significantly elevated rates of MDD (46.0%) relative to their co-twin who did not have CUD (28.12%; OR 2.83, 95% CI 1.12–7.19; N = 63 MZ pairs).

The model-fitting results are summarized in Table 3. In addition to the 13 co-morbidity models, we included a saturated model based on twin correlations for comparison. Five models can be rejected, due to the large, statistically significant differences between the observed cell counts within the co-morbidity categories (see Table 2) and the cell counts expected under the model: the *Chance, Alternate Forms, Three Independent Disorders, RM of MDD*, and *EM of MDD* models.

**Table 3:** Co-morbidity model fit statistics and questions models aim to address

Model		Question	χ2	Df	p	AIC
Saturated Model			13.16	11	0.283	−8.84
1	Alternate Forms	Alternate forms of the same disorder?	96.31	14	<0.001	68.31
2	Three Indep. Disorders	Co-morbid form is an independent disorder?	32.90	8	<0.001	16.90
3	Random Multiformity	Abruptly increase symptoms of each other?	15.10	10	0.129	−4.90
4	RM of MDD	MDD abruptly increases CUD symptoms?	27.04	11	0.004	5.04
5	RM of CUD	CUD abruptly increases MDD symptoms?	15.46	11	0.162	−6.54
6	Extreme Multiformity	Abruptly increase symptoms of each other in extreme cases?	16.32	10	0.091	−3.68
7	EM of MDD	MDD abruptly increases CUD symptoms in extreme cases?	28.23	11	0.003	6.23
8	EM of CUD	CUD abruptly increases MDD symptoms in extreme cases?	19.50	11	0.053	−2.50
9	Correlated Liabilities	Liabilities are correlated?	15.21	9	0.085	−2.79
10	Reciprocal Causation	CUD and MDD cause each other?	15.23	10	0.124	−4.77
11	MDD causes CUD	MDD causes CUD?	17.86	11	0.085	−4.14
12	CUD causes MDD	CUD causes MDD?	15.50	11	0.161	−6.50
13	Chance	Co-morbid due to chance?	59.46	12	<0.001	35.46

**Fig. 1:**
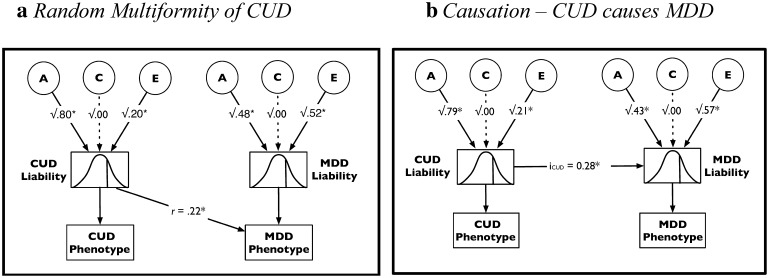
Parameter estimates from best fitting co-morbidity models: **a** *Random Multiformity of CUD*, **b** *Causation—CUD causes MDD. r* probability of MDD phenotype if above threshold on CUD liability, *i*_*CUD*_ regression coefficient, *Significant at the 0.05 level

The only models that do not have substantially less support than the saturated model (i.e. an AIC difference larger than 3) are the *RM of CUD* (model 5) and *CUD causes MDD* (model 12) models (see Fig. 1a, b), with differences of 2.30 and 2.34 respectively. Both models have substantially more support than the *Correlated Liabilities* model.

These two best fitting models are, however, not substantially different from some of the models within their class. The *RM of CUD* model is not substantially different from the *Random Multiformity* model. The *CUD causes MDD* model is not substantially different from the *MDD causes CUD* and *Reciprocal Causation* model. Additionally, both models do not substantially differ from the *Extreme Multiformity* model.

In the best fitting models both CUD and MDD are influenced by genetic and non-shared environmental factors. In the case of CUD, 79–80% of the total variance is estimated to be explained by genetic factors and 20–21% by non-shared environmental factors. For MDD, 43–48% of the total variance is explained by genetic, 52–57% by non-shared environmental factors. Model-fit did not significantly deteriorate when C was dropped from both models. Parameter estimates from all models can be found in Table 4.

**Table 4:** Parameter estimates of all co-morbidity models for MDD and CUD

	Model												
	1	2	3	4	5	6	7	8	9	10	11	12	13
a^2^_MDD_	–	0.46(0.23–0.56)	0.48(0.20–0.60)	0.46(0.23–0.57)	0.48(0.19–0.60)	0.48(0.17–0.59)	0.46(0.23–0.56)	0.48(0.17–0.59)	0.45(0.18–0.56)	0.44(0.19–0.55)	0.47(0.22–0.57)	0.43(0.18–0.54)	0.46(0.23–0.56)
c^2^_MDD_	–	0.00(0.00–0.17)	0.00(0.00–0.22)	0.00(0.00–0.17)	0.00(0.00–0.23)	0.00(0.00 – 0.35)	0.00(0.00–0.45)	0.00(0.00–0.08)	0.01(0.00–0.21)	0.00(0.00–0.14)	0.00(0.00–0.19)	0.00(0.00–0.18)	0.00(0.00–0.18)
e^2^_MDD_	–	0.54(0.44–0.66)	0.52(0.40–0.65)	0.54(0.43–0.66)	0.52(0.40–0.64)	0.52(0.41–0.65)	0.54(0.44–0.66)	0.52(0.41–0.65)	0.56(0.44–0.67)	0.56(0.45–0.70)	0.53(0.43–0.65)	0.57(0.46–0.70)	0.54(0.44–0.66)
a^2^_CUD_	–	0.76(0.45–0.87)	0.82(0.52–0.90)	0.86(0.53–0.94)	0.80(0.50–0.87)	0.74(0.41–0.83)	0.76(0.45–0.87)	0.74(0.41–0.83)	0.79(0.48–0.87)	0.80(0.48–0.88)	0.81(0.52–0.88)	0.79(0.79–0.87)	0.76(0.45–0.87)
c^2^_CUD_	–	0.03(0.00–0.29)	0.00(0.00–0.24)	0.03(0.00–0.31)	0.00(0.00–0.24)	0.01(0.00–0.28)	0.03(0.00–0.29)	0.01(0.00–0.28)	0.01(0.00–0.26)	0.00(0.00–0.03)	0.00(0.00–0.23)	0.00(0.00–0.26)	0.03(0.00–0.29)
e^2^_CUD_	–	0.21(0.13–0.31)	0.18(0.10–0.29)	0.12(0.06–0.21)	0.20(0.13–0.31)	0.25(0.17–0.36)	0.21(0.13–0.31)	0.25(0.17–0.36)	0.20(0.13–0.31)	0.20(0.12–0.30)	0.19(0.12–0.30)	0.21(0.13–0.32)	0.21(0.13–0.31)
a^2^_shared_	0.66(0.66–0.66)	0.53(0.00–1.00)	–	–	–	–	–	–	0.19(–0.03–0.31)	–	–	–	–
c^2^_shared_	0.34(0.34–0.34)	0.02(0.00–1.00)	–	–	–	–	–	–	0.01(−.07–0.17)	–	–	–	–
e^2^_shared_	0.00(0.00–0.00)	0.45(0.00–1.00)	–	–	–	–	–	–	0.08(−0.01–0.17)	–	–	–	–
*p*	0.44(0.44–0.44)	–	0.02(0.02–0.08)	0.10(0.06–0.14)	–	–	–	–	–	–	–	–	–
*r*	0.27(0.27–0.27)	–	0.20(0.09–0.27)	–	0.22(0.15–0.29)	–	–	–	–	–	–	–	–
i_MDD_	–	–	–	–	–	–	–	–	–	07(−0.19–0.32)	0.26(0.18–0.35)	–	–
i_CUD_	–	–	–	–	–	–	–	–	–	21(−0.05–0.52)		0.28(0.20–0.38)	
t_MDD_	–	0.63	0.71	0.64	0.72	0.69; 2.21	0.64; 1.92	0.72	0.64	0.66	0.64	0.66	0.63
t_CUD_	–	0.96	1.01	1.06	0.99	1.04; 1.98	1.07	1.00; 1.81	0.99	1.01	1.03	.0.99	0.96
t_shared_	−0.31	1.13	–	–	–	–	–	–	–	–	–	–	–

Model numbers refer to co-morbidity models outlined in Table 1. Shared a^2^, c^2^ and e^2^ refer to the single shared liability in model 1, third independent liability in model 2, or Cholesky paths from MDD to CUD in model 9

*p* probability of CUD if above threshold on the MDD liability (models 3–5), or above threshold on the shared liability (model 1), *r* probability of MDD if above threshold on the CUD liability (models 3–5), or above threshold on the shared liability (model 1), *i*_*CUD*_ regression coefficient from CUD to MDD, *i*_*MDD*_ regression coefficient from MDD to CUD, *t*_*MDD*_ threshold for MDD, *t*_*CUD*_ threshold for CUD

## Discussion

To our knowledge, this study is the first to fit the 13 co-morbidity models proposed by Neale and Kendler (1995) to cannabis use disorder and major depressive disorder. Both epidemiological and discordant twin analyses confirmed that these models were appropriate for the current sample. In line with other cross-sectional studies (see e.g. Degenhardt et al. 2012) epidemiological analyses showed that CUD and MDD were significantly co-morbid. Discordant twin analyses, which will be discussed further below, showed that causal processes could not be excluded as an explanation for this co-morbidity, because MZ twins with CUD were significantly more likely to display symptoms of MDD than their co-twin without CUD. Therefore, there was sufficient reason to further explore causality in the co-morbidity model analyses.

The two best-fitting models were *Random Multiformity of CUD* and *CUD causes MDD*. Both models fit substantially better than the *Correlated Liabilities* model, and not substantially worse than the *Saturated* model. In addition, five models could be statistically rejected: the *Alternate Forms, Chance, Three Independent Disorders, RM of MDD* and *EM of MDD* models. The heritability estimates in the best fitting models range from 79 to 80% for CUD and 43 to 49% for MDD.

### Model-fitting

These model-fitting results suggest that the direction of effect goes from CUD to MDD. Firstly, both *RM of MDD* and *EM of MDD* can be statistically rejected. It seems plausible, therefore, that the fit of the bi-directional *Random Multiformity* and *Extreme Multiformity* models is driven by the paths they have in common with *RM of CUD* and *EM of CUD*, respectively. Secondly, the *CUD causes MDD* fits better than the *MDD causes CUD* model. Although this difference is not substantial, the fit of the *MDD causes CUD* model may reflect that *Direction of Causation* models are difficult to distinguish when modes of inheritance of the disorders are similar (Heath et al. 1993). In the current study, this may be because both disorders are mainly influenced by A and E, rather than by different etiological factors (e.g. A C E vs. A E). Lastly, the *MDD causes CUD* model, along with all other models with a direction of effect from MDD to CUD, was a substantially poorer fit than the *Saturated Model*.

It is unclear, however, which of the two best-fitting models is more likely. The *CUD causes MDD* model assumes that the liability to develop MDD symptoms increases *continuously*, as the risk of CUD increases. The threshold in this model does not equal a sudden increase in risk, which means that even sub-threshold increases in liability to CUD have a causal influence on the liability to develop MDD (Rhee et al. 2004). On the other hand, the *RM of CUD* model assumes that the risk of MDD symptoms increases *discontinuously*, once the threshold on the CUD liability has been passed (i.e. an individual has reached a liability high enough to develop the disorder). An additional difference between the models is their assumption about etiological processes. The causal model assumes that any causal processes occur at the level of the liability (Rhee et al. 2004), while the RM models remain agnostic about the way in which one disorder leads to symptoms of the other.

Despite some differences, the *RM of CUD* and *CUD causes MDD* models are not incompatible. Causality may play a role, and the good fit of the *RM of CUD* model may indicate that the causal influences on the risk of MDD only occur at higher levels of CUD risk (i.e. post-threshold). Additionally, it is likely that there are shared etiological factors between CUD and MDD. Evidence from twin (Fu et al. 2002; Lynskey et al. 2004) and molecular genetic studies (Bobadilla et al. 2013; Sherva et al. 2016; Hodgson et al. 2016) suggests that there are genetic factors influencing both cannabis involvement and MDD. There is also a plethora of environmental factors that act as risk factors for both (e.g. Fergusson and Horwood 1997; Feingold et al. 2014). Overall, the almost identical fit of both models may indicate that there are threshold-dependent causal links from CUD to MDD, which occur at the level of liability.

This interpretation is compatible with several findings. Risk factors for CUD, such as heavy cannabis use, are likely to exert an environmental and genetic effect on MDD. Heavy cannabis use can alter various domains of cognitive functioning, such as attention and memory (Solowij 2002), and thereby affect daily functioning and potentially create circumstances in which individuals are more likely to develop MDD. For instance, cannabis use impacts negatively on educational attainment (Lynskey and Hall 2000), which in turn may affect emotional wellbeing. Environmental effects may also manifest themselves through changes in brain structure and function. Heavy cannabis users show a decrease in amygdala volumes (Yucel et al. 2008), which is also the case in un-medicated patients with MDD (Hamilton et al. 2008). Furthermore, the endocannabinoid system, primary site of the neurochemical effects of cannabis, is thought to be involved in mood regulation (Ashton and Moore 2011). Genes may modulate these environmental influences. Lastly, the conclusion that causal processes may be at work in individuals at high risk for CUD (e.g. high levels of cannabis use), also fits well with longitudinal studies which show that high levels of cannabis use are more strongly associated with MDD than lower levels.

### Heritability estimates

The heritability estimates obtained from the models are similar to other twin studies for MDD (Sullivan et al. 2000; Kendler et al. 2006b), and to studies on cannabis abuse/dependence that included similar samples. Kendler et al. (2006a) report a heritability estimate of 77% (95% CI 46–93%) for DSM-IV cannabis abuse/dependence in a sample of same-sex and opposite-sex twins with a mean age of 28.2. While a meta-analysis on twin studies reporting at least 1 symptom of abuse/dependence, presents lower heritability estimates [males: 54.4% (95% CI 37.9–64.9%), females: 58.5 (95% CI 44.2%–72.9%), Verweij et al. 2010], the higher estimate obtained in the current may be related to differences in sampling or the definition of problematic cannabis use.

### Limitations and future research

Difficulties in differentiating between models were a known limitation, based on previous studies. Rhee et al. (2004) provide a detailed discussion of general limitations of the NK model fitting approach. Although Rhee et al. (2004) demonstrated that the NK approach to discriminating between different models of co-morbidity is valid; they did so with a large simulated sample and still noted several challenges. They highlighted that it is particularly difficult to discriminate between the *multiformity* and the *correlated liabilities* model classes, which was also the case in the current sample. Additionally, Rhee et al. (2004) pointed out that discrimination within subclasses of models (e.g. *RM* vs. *RM of CUD*) is also problematic. In the current analyses, the difference within subclasses was often not more than 3 AIC. It may be beneficial to replicate the study with larger samples or use meta-analysis to examine whether differences between models become more distinct. Replication of our results would be useful to explore whether the results of the current study are cohort-specific or generalize across cohorts, but is outside the scope of the present study.

One limitation of the current study is that sex differences have not been taken into account. The prevalence of MDD and CUD did differ between males and females in the analysis sample, but currently all co-morbidity models can only be fitted on contingency tables, in which it was not possible to specify separate thresholds for males and females. The alternative approach of fitting separate models for males and females was not feasible due to lack of power. However, there are currently no grounds to assume that different co-morbidity models would explain co-morbid cases in males and females. For instance, Agrawal et al. (2010) examined the co-morbidity between cannabis and tobacco use, and fitted separate models for male and female twins. They found that model fits were very similar for both sexes. It may be an interesting avenue for future research to explore sex differences in larger samples or using meta-analysis.

Given that one of the best-fitting models makes assumptions about causality, it is also an important limitation that the data are retrospective and age of onset was not considered in the analyses. Using retrospective data has several disadvantages (see e.g. Coughlin 1990), but for the current analyses the most pertinent drawback is that longitudinal data would be better suited to test direction of causation. Beyond twin models, recent molecular genetic methods also offer an interesting avenue to assess causality (see Pickrell et al. 2016).

Finally, the discordant twin results are difficult to interpret due to the small number of MZ twin pairs discordant for CUD. The results are in contrast to study results in Lynskey et al. (2004) and in line with those in Lin et al. (1996), but overall they are not entirely comparable to either: both studies examined cannabis dependence rather than CUD. To conclusively examine whether causal processes can be excluded using the discordant twin method, larger sample sizes would be necessary. However, for the purposes of the current study, the main intention was establishing that causal processes could not be ruled out within the current data set.

A valuable next step would be the inclusion of known confounding factors. As mentioned above, Fu et al. (2002) have found that antisocial personality disorder, while being comparatively rare (Coid et al. 2006) and therefore unlikely to explain most co-morbid cases, is a significant confounder in the genetic relationship between cannabis dependence and MDD. Moreover, longitudinal studies have highlighted that cannabis users and non-users differ on a number of domains (Fergusson and Horwood 1997; Feingold et al. 2014). As such, it would be valuable to examine which models provide the best fit when confounding factors are included.

## Conclusion

Overall, the model fitting approach has been a beneficial indicator of the likely relationship between CUD and MDD. While it was not possible to statistically differentiate between the two best fitting models *RM of CUD* and *CUD causes MDD*, they both seem to indicate that the direction of influence goes from CUD to MDD. Combined, the models suggest that CUD risk factors may cause MDD symptoms, but only in higher risk individuals. In addition, several models can be statistically excluded: CUD and MDD are not likely to be co-morbid by chance, arise from the same risk factors, or be due to a liability separate from the pure form of the disorders. The fact that a *Random Multiformity* model is the best fitting model is remarkable, because this model is not widely reported. Replications on larger samples would be beneficial in order to help differentiate between models with subtle differences.
